# Transcriptome Analysis Reveals Potential Mechanism in Storage Protein Trafficking within Developing Grains of Common Wheat

**DOI:** 10.3390/ijms232314851

**Published:** 2022-11-27

**Authors:** Zeeshan Ali Buttar, Abdullah Shalmani, Mohsin Niaz, Chaojie Wang, Shahid Hussain, Chengshe Wang

**Affiliations:** 1 State Key Laboratory of Crop Stress Biology in Arid Areas, College of Agronomy, Northwest A & F University, Xianyang 712100, China; 2 State Key Laboratory of Wheat and Maize Crop Science, College of Agronomy, Center for Crop Genome Engineering, Longzi Lake Campus, Henan Agricultural University, Zhengzhou 450046, China; 3 College of Life Sciences, Northwest A & F University, Xianyang 712100, China; 4 CIMMYT-China Joint Center of Wheat and Maize Improvement, National Key Laboratory of Wheat and Maize Crop Science, Agronomy College, Henan Agricultural University, Zhengzhou 450046, China; 5 Jiangsu Key Laboratory of Crop Genetics and Physiology, Jiangsu Key Laboratory of Crop Cultivation and Physiology, Jiangsu Co-Innovation Center for Modern Production Technology of Grain Crops, Research Institute of Rice Industrial Engineering Technology, Yangzhou University, Yangzhou 225009, China

**Keywords:** wheat (*Triticum aestivum* L.), grain storage protein (gluten), gene expression

## Abstract

Gluten proteins are the major storage protein fraction in the mature wheat grain. They are restricted to the starchy endosperm, which defines the viscoelastic properties of wheat dough. The synthesis of these storage proteins is controlled by the endoplasmic reticulum (ER) and is directed into the vacuole via the Golgi apparatus. In the present study, transcriptome analysis was used to explore the potential mechanism within critical stages of grain development of wheat cultivar “Shaannong 33” and its sister line used as the control (CK). Samples were collected at 10 DPA (days after anthesis), 14 DPA, 20 DPA, and 30 DPA for transcriptomic analysis. The comparative transcriptome analysis identified that a total of 18,875 genes were differentially expressed genes (DEGs) between grains of four groups “T10 vs. CK10, T14 vs. CK14, T20 vs. CK20, and T30 vs. CK30”, including 2824 up-regulated and 5423 down-regulated genes in T30 vs. CK30. Further, the Kyoto Encyclopedia of Genes and Genomes (KEGG) pathway enrichment highlighted the maximum number of genes regulating protein processing in the endoplasmic reticulum (ER) during grain enlargement stages (10–20 DPA). In addition, KEGG database analysis reported 1362 and 788 DEGs involved in translation, ribosomal structure, biogenesis, flavonoid biosynthesis pathway and intracellular trafficking, secretion, and vesicular transport through protein processing within ER pathway (ko04141). Notably, consistent with the higher expression of intercellular storage protein trafficking genes at the initial 10 DPA, there was relatively low expression at later stages. Expression levels of nine randomly selected genes were verified by qRT-PCR, which were consistent with the transcriptome data. These data suggested that the initial stages of “cell division” played a significant role in protein quality control within the ER, thus maintaining the protein quality characteristics at grain maturity. Furthermore, our data suggested that the protein synthesis, folding, and trafficking pathways directed by a different number of genes during the grain enlargement stage contributed to the observed high-quality characteristics of gluten protein in Shaannong 33 (*Triticum aestivum* L.).

## 1. Introduction

Bread wheat (*Triticum aestivum* L.) is one of the main sources of protein (20%) and dietary calories for 2.5 billion of the world’s population [1,2,3,4,5]. The annual production of wheat is 722.4 million metric tons in an area of 220 million hectares worldwide, making it one of the dominant cereal crops [6,7,8]. The two species, *Triticum aestivum* and *Triticum turgidum* var durum, represent a significant contribution to global wheat production. However, the hexaploid *T. aestivum* or common bread wheat accounts for 95% in global wheat production for bread making [9,10]. Further, distinguished properties of wheat flour provide opportunities to process it into different end products, such as steamed bread, noodles, pasta, cookies, and dumplings [11,12,13,14,15,16]. Thus, the end product quality of wheat is determined by gluten proteins, comprised of polymeric glutenins and monomeric gliadins. The gliadins and glutenin have intermolecular disulfide bonds shaped into gluten complexes, which determine the dough viscoelasticity during flour processing [17,18], among which glutenins offer strength and elasticity [19,20], whereas gliadins offer viscosity to the dough [20,21,22]. Among all the constituents, grain storage protein (GSP) or “Gluten” plays a significant role in the high-quality end product. Therefore, selection of cultivated wheat by grain and yield traits has shaped the unique features of wheat cultivars. However, the potential mechanism that controls the underpinning mechanism of GSP within the developing grain is still mystery.

Wheat genotypes vary in native glutenin subunit composition, influencing dough quality and determining the suitability of the genotype for any specific end-products. There are four classes of wheat proteins, namely albumin, globulin, gliadin, and glutenins [21,22,23,24]. The extremely strong flour confers so much elasticity that the expansion of dough is reduced, thus resulting in poor-quality bread but good-quality noodles. Most importantly, the accumulation and expression of gluten protein taking place within developing grains accumulate to relatively high amounts in the endospermic tissues [25,26]. However, the expression of different transcription factors associated with synthesis and trafficking during grain enlargement is not known. Specifically, at different development stages, in developing endosperm, the seed GSP is transported from the rough endoplasmic reticulum (ER) to Golgi bodies using two distinct routes: one comprises the vesicle bud direct to the ER, while the other transports through Golgi [27,28,29,30,31]. Previous successful work has demonstrated that different clades of genes mediate this transport and represent a significant contribution to altering the processing quality of wheat [32,33]. Further, a combination of genotype and cultivation environment plays a pivotal role in defining the structure properties of GSPs [32,33,34,35,36]. Therefore, the ER works as an entry port of protein and the quality control function during transport to the Golgi complex [28,29,37,38,39,40]. Further, it controlled the function of protein quality by synthesis and folding. Therefore, it is still a matter of interest to understand the specific stage and underlying mechanism of the ER protein quality control mechanism in high-quality wheat cultivars.

Next-generation sequencing (NGS) is rapidly gaining ground. It has provided an important platform for genome-wide transcriptional profiling [41,42,43,44,45,46,47,48,49]. In wheat, due to the occurrence of multiple copies of gene sequences (homologous or paralogous genes), sequence assembly and annotation seem very daunting [43,44,46,47,50]. Transcriptomic profiling of the developing grain has provided a novel approach to increase the understanding of the biology of the developing grain in association with candidate genes [42,43,44,45,46,47,48,49,50,51]. Previous studies have reported three stages of gene expression within the developing wheat grain [52,53,54,55]. The first major transition stage is within 10 days post anthesis (DPA): “extensive cell division, expansion, and differentiation”, to make milky endosperm and embryo [48,56,57]. The second major transition stage remains until 20 DPA, accumulating “starch and seed storage proteins” within cells to make semi-solid endosperm with optimum transcriptional activities, occurring at 14 DPA [53,54,56,57]. The third transition starts at 30 DPA through the deposition of storage reserves [56,57,58]. The present study used Shaannong-33 acknowledged as the first extra-strong gluten wheat cultivar released in China, reported tensile resistance valued 1038 E.U, introduced in Henan Province, while the sister line of Shaannong-33 was reported with one third of its maximum tensile resistance, provided in detail in Table 1. The samples were collected at four important stages, 10 DPA, 14 DPA, 20 DPA, and 30 DPA, of the developing grain, to study crucial stages of the ER protein quality control mechanism and identify new candidate genes within developing grains.

## 2. Results

### 2.1. Sequence Assembly Based on cDNA Libraries

**Figure 1:**
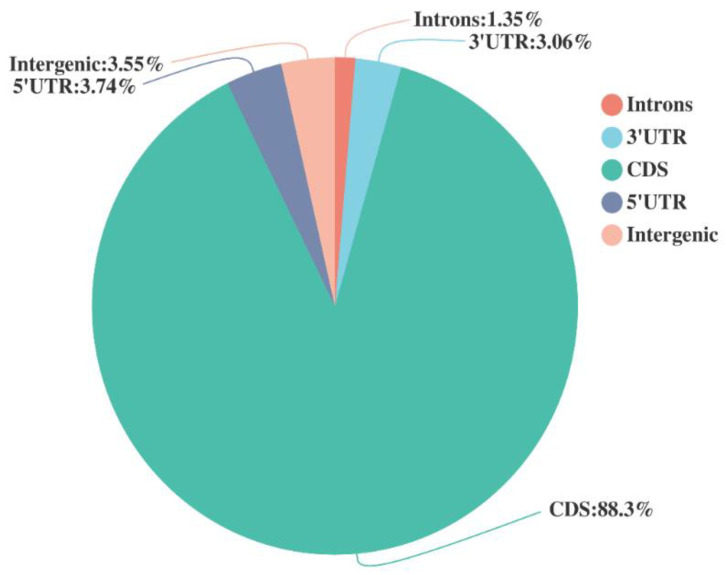
Pie charts showing the percentage of different repeated elements identified in wheat genome of collected samples. The most abundant components identified were CDS, intergenic, introns, and 3’UTR: 3.06% followed by 5’UTR.

**Figure 2:**
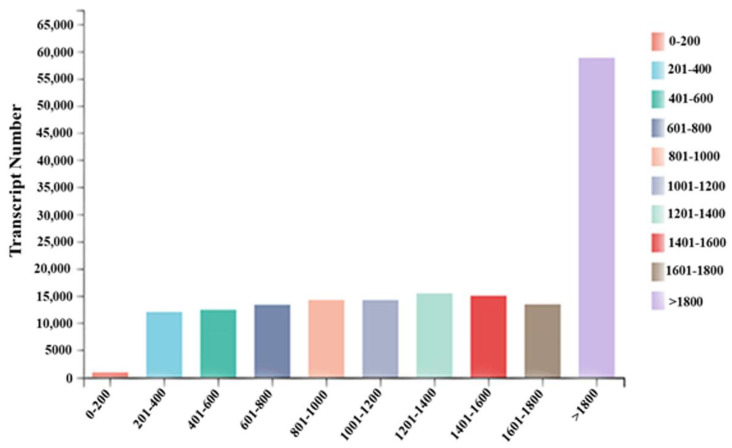
The length distribution of reads of transcripts length. The length and distribution of reads aligning to the reference genome *Triticum_aestivum*, version: iwgsc_refseqv1.0 source, from selected 0–200 (brown), 201–400 (Cyan), 401–600 (Aqua), 601–800 (Cadet blue), 801–1000 (Light salmon), 1001–1200 (Light steel blue), 1201–1400 (Aquamarine), etc.

A total of 51,306,098, 57,143,490, 48,289,034, 48,697,252, of 10 DPA (T10), 14 DPA (T14), 20 DPA (T20), 30 DPA (T30) and 51,814,526, 51,232,492, 48,113,392, and 50,053,338 respectively reported clean reads libraries of CK10, CK14, CK20, and CK30. The raw reads consisted of 52,242,026, 58,357,186, 49,861,908, 49,553,172, 52,674,730, 52,215,230, 48,982,074, and 51,353,948 of T10, T14, T20, T30, and CK10, CK14, CK20, CK30, respectively. The raw bases showed 788,8545,926, 8,811,935,086, 7,529,148,108, and 7,482,528,972 at T10, T14, T20, and T30, respectively. GC content, Q20, and Q30 were 54.64%, 98.03%, and 94.4% for T10; 53.14%, 97.75%, and 93.89% for T14; 52.91%, 97.17%, and 92.6% for T20; 54.35%, 97.96%, and 94.25% for T30, respectively. Similarly, CK10 showed 55.02, 98.11, and 94.59 GC, Q20, and Q30 respectively; further detail is given in Table 2. Q20 and Q30 express the chance of error, with >97% confidence and <3% chance of error, respectively. Therefore, all the data represent the suitability to be used for downstream analysis. Further, the grain transcript reads for all collected samples are showed in Figure 1. The transcript numbers between 0–200, 201–400, 401–600, and 601–800 were 804, 12,014, 12,376, and 13,310, respectively, with complete detail in Figure 2 and Supplementary File S5, sheet 1. There were eight library constructions for all collected samples (T10, T14, T20, and T30 and CK10, CK14, CK20, and CK30) used for the current study, showing >85% CDS reads. The transcription length reported within the used sample is given in Figure 2 for comprehensive analysis of Shaannong 33 (*Triticum aestivm* L.) and CK. Further, we divided all collected stages into four groups (T10 vs. CK10, T14 vs. CK14, T20 vs. CK20, and T30 vs. CK30).

**Figure 3:**
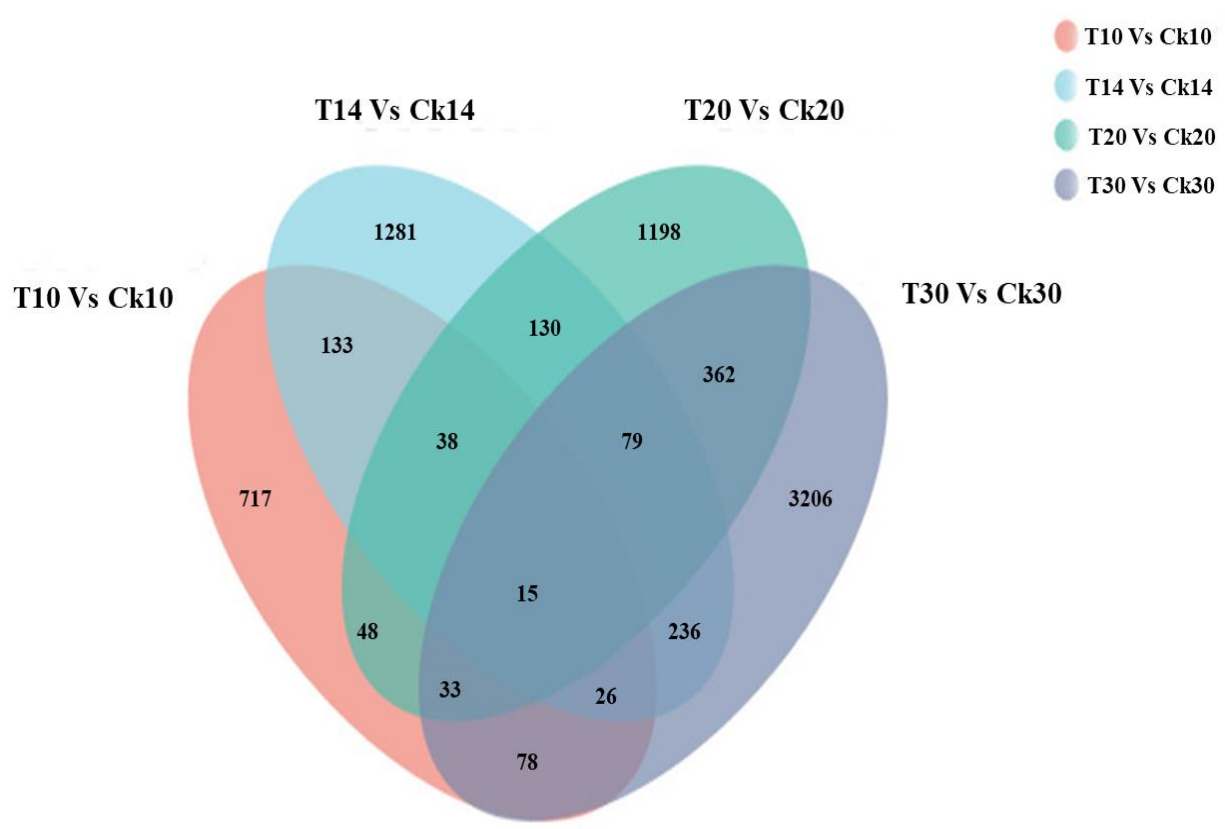
Venn diagram showing the number of common and unique DEGs among four colored group samples. Dark gray group shows the highest number of DEGs and shares more DEGs with the other three groups in comparison to red, blue, and green groups.

**Figure 4:**
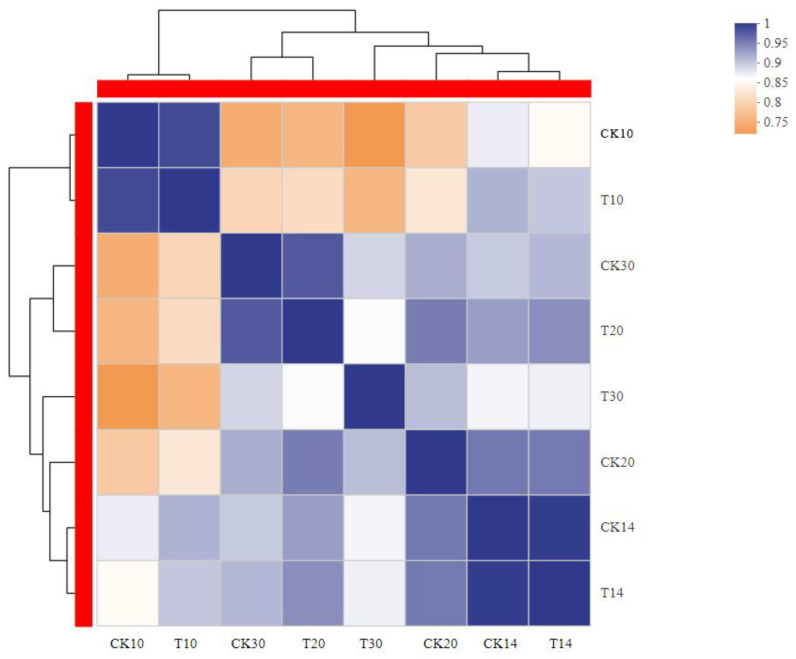
Correlation heat map for all 8 samples. The heat map plots the correlation coefficient score between any two samples. Samples #10, #14, #20, and #30 were clear outliers relative to the other samples.

A total of 717, 1281, 1198, and 3206 were DEGs for all four groups (T10 vs. CK10, T14 vs. CK14, T20 vs. CK20, and T30 vs. CK30), respectively, given in Figure 3. The maximum similarity index of 14% was between “T10 vs. Ck10” and “T20 vs. CK20”. Similarly, the lowest similarity index was 5.3% for “T10 vs. Ck10” and “T14 vs. CK14” in Figure 3. Further, the heatmap of all collected samples (T10, T14, T20, T30, CK10, CK14, CK20, and CK30) correlated, based on the samples tested at different stages (Figure 4 detail shown in Supplementary File S5, sheet 2).

### 2.2. Functional Annotation between Different Grain Development Stages

**Figure 5:**
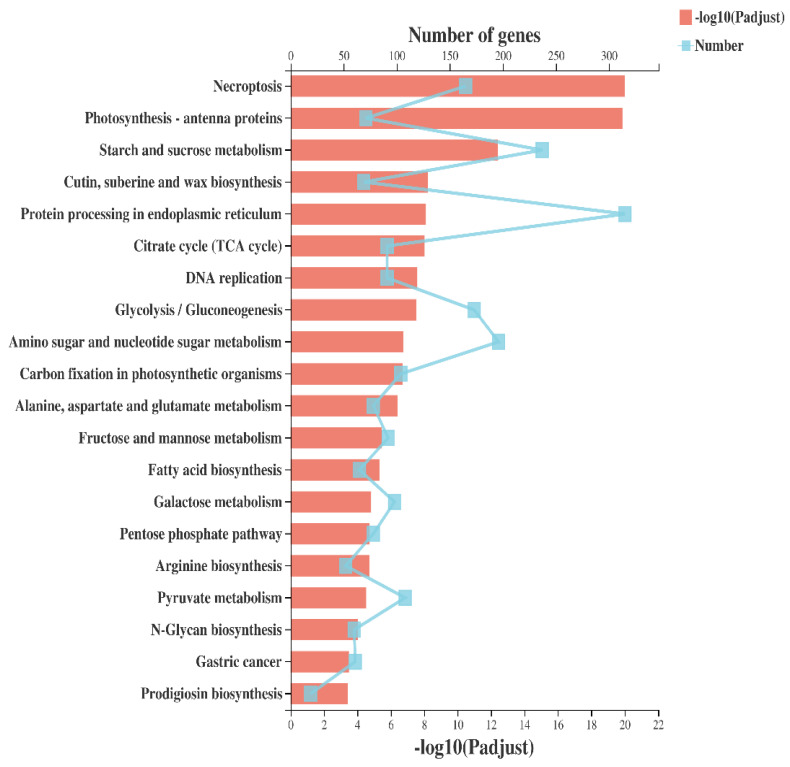
KEGG pathway enrichment analysis for 127 candidate genes. The x-axis shows the gene ratio; the y-axis corresponds to KEGG pathways. The dot color represents the corrected P value of <0.05, and the dot size represents the number of genes enriched in the reference pathway. The protein processing in endoplasmic reticulum, starch and sucrose metabolism, amino sugar and nucleotide sugar metabolism, glycolysis/gluconeogenesis, necroptosis, and pyruvate metabolism was significantly enriched, detail given in Supplementary File S1, sheet 1.

**Figure 6:**
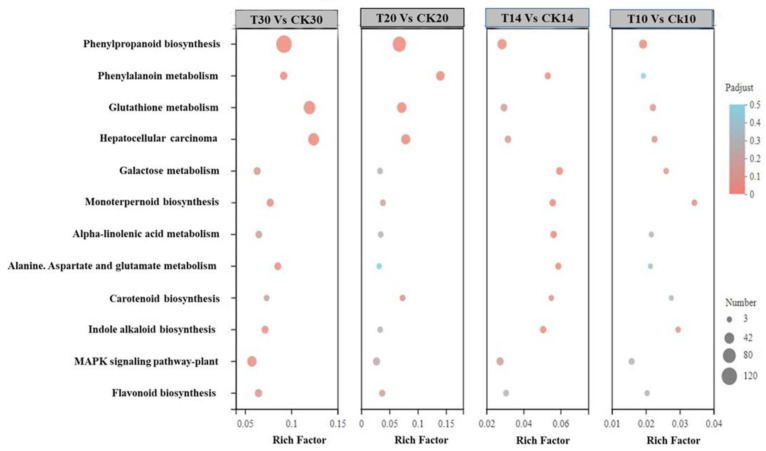
Clusters of KEGG function classification of DEGs based on significantly enriched KEG terms are indicated by dot for corrected *p* value of <0.05. The dot size represents the number of genes enriched in the reference pathway. There were a total of 108, 115, 99, and 104 DEGs for 715, 697, 366, and 1535 pathways in (T10 vs. CK10, T14 vs. CK14, T20 vs. CK20, and T30 vs. CK30), respectively. Detail given in Supplementary File S2.

**Figure 7:**
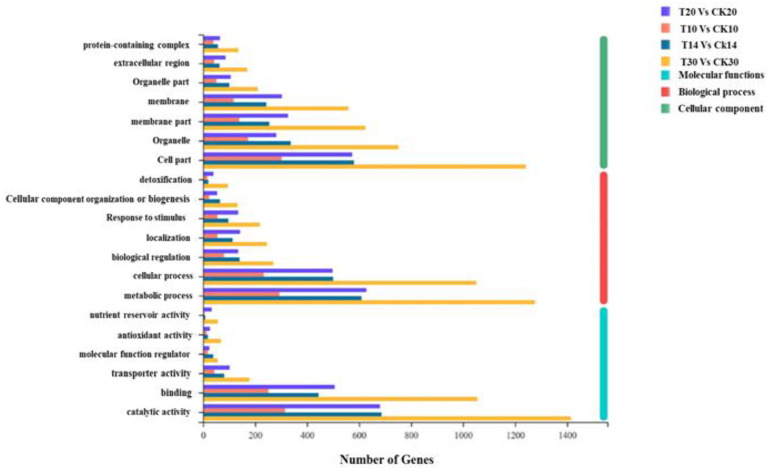
Histogram of Gene Ontology (GO) classifications of DEGs between T10 vs. CK10, T14 vs. CK14, T14 vs. CK14, T20 vs. CK20, and T30 vs. CK30. A total of 373, 378, 203, and 444 DEGs were assigned to three main GO functional categories and then divided into 20 sub-categories.

**Figure 8:**
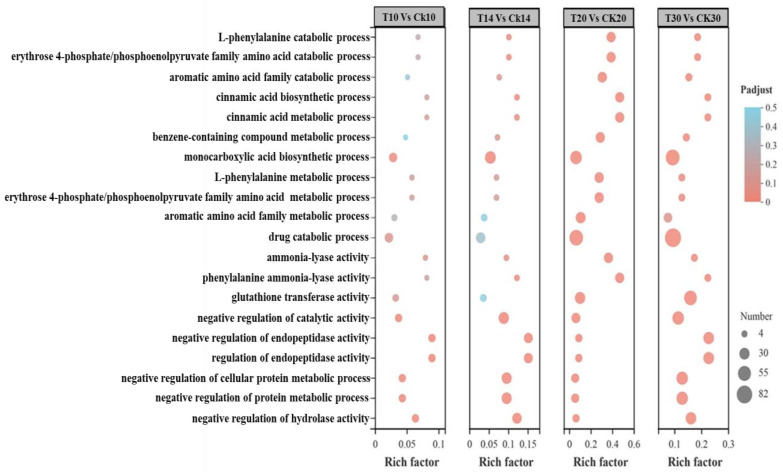
Clusters of GO function classification of DEGs. A total of 1920 DEGs were mapped to 107 KEGG pathways. There were 373, 379, 444, and 203 DEGs for (T10 vs. CK10, T14 vs. CK14, T20 vs. CK20, and T30 vs. CK30), respectively.

First, KEGG enrichment analysis was conducted by using DEGs at all four stages and libraries of all collected samples (T10, T14, T20, and T30 and CK10, CK14, CK20, and CK30), as shown in Figure 5 and Supplementary File S1 sheet 2. The maximum functional annotation of 315 DEGs in pathway id (map04141) is linked with protein processing within the ER; that in the secondary category is described as folding, sorting, and degradation of GSPs; 237 DEGs were observed in starch and sucrose metabolism in pathway id (map00500); 213 in pathway id map04075 were associated with plant hormone signal transduction. Further, the KEGG enrichment analysis was conducted by using all four groups (T10 vs. CK10, T14 vs. CK14, T20 vs. CK20, and T30 vs. CK30), provided in Figure 6 in detail, Supplementary File S1 (sheets 1–4). The rich factor with Padjust (0–0.5) showed phenylpropanoid biosynthesis and carotenoid biosynthesis, of which the pathway id was “map00940”. There were 8, 3, 8, and 8 numbers of genes in association with pathway map00906 in all four groups of (T30 vs. CK30, T20 vs. CK20, T14 vs. CK14, and T10 vs. CK10). For further improvement of our knowledge about the total number of genes involved in grain development, function classification of clusters of orthologous groups (COGs) was conducted for all four groups. There was a distribution of 20 COG categories based on COG functional classification, shown in Figure 7. The cell part involved of 1200 genes in group “T30 vs. CK30”, in comparison with >200 DEGs involved in protein processing of “T30 vs. CK30” of cellular components. Furthermore, a functional description is given in Supplementary File S1, sheet 3. A total of 1362 DEGs were reported in translation, ribosomal structure, and biogenesis in “T14 vs. CK14”, in comparison with other groups. Consistent with functional analysis, we performed GO enrichment analysis of all four groups (T10 vs. CK10, T14 vs. CK14, T20 vs. CK20, and T30 vs. CK30), as shown in Figure 8 and Supplementary File S3, sheets 1–4. There were 20 categories of functional classification, comprised of negative regulation of the protein metabolic process, negative regulation of the cellular protein metabolism process, and so on, with further detail given in Figure 8 and Supplementary File S3.

### 2.3. Pathway Analysis of Protein Processing in Endoplasmic Reticulum (ER)

**Figure 9:**
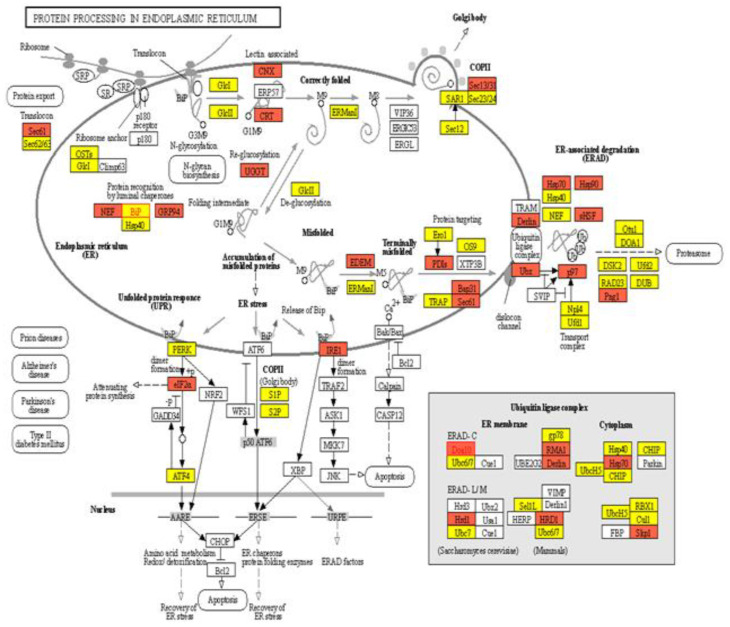
KEGG pathway analysis identified the ER-associated protein trafficking pathway (ko04141) for DEGs between 10 DPA, 14 DPA, 20 DPA, and 30 DPA. Red, yellow, and white indicate genes expression increase, decrease, and mixed change at all selected 10, 14, 20, and 30 DPA, respectively.

The synthesis, folding, and deposition of the gluten proteins take place within the endomembrane system of a developing grain. They fold in the lumen or membrane of the ER from where they are sorted toward their site of action [26,58,59]. ER protein quality control in the biological processes has long been a subject of intensive study. In the present study, KEGG pathway analysis identified 7, 9, 19, and 17 DEGs for the cellular amino acid metabolic process, protein metabolic process, Golgi vesicle transport, and protein serine/threonine kinase activity, respectively, in association with protein processing in the ER pathway (ko04141) (Figure 9), and for the protein export pathway (map03060) (Figure S1). The protein quality is controlled within the ER. The protein synthesis, folding, and trafficking are marked by the composition of N-glycan. Here, we observed that the folding sensor UGGT acts as an unusual molecular chaperone and covalently modifies (G1M9) the folding intermediate to correctly folded M9 ERMan1. The putative genes take part in recognition of folding and misfolded proteins within the ER; in addition, genes are listed in Table 3.

### 2.4. Analysis of Different Groups of Differently Expressed Genes

**Figure 10:**
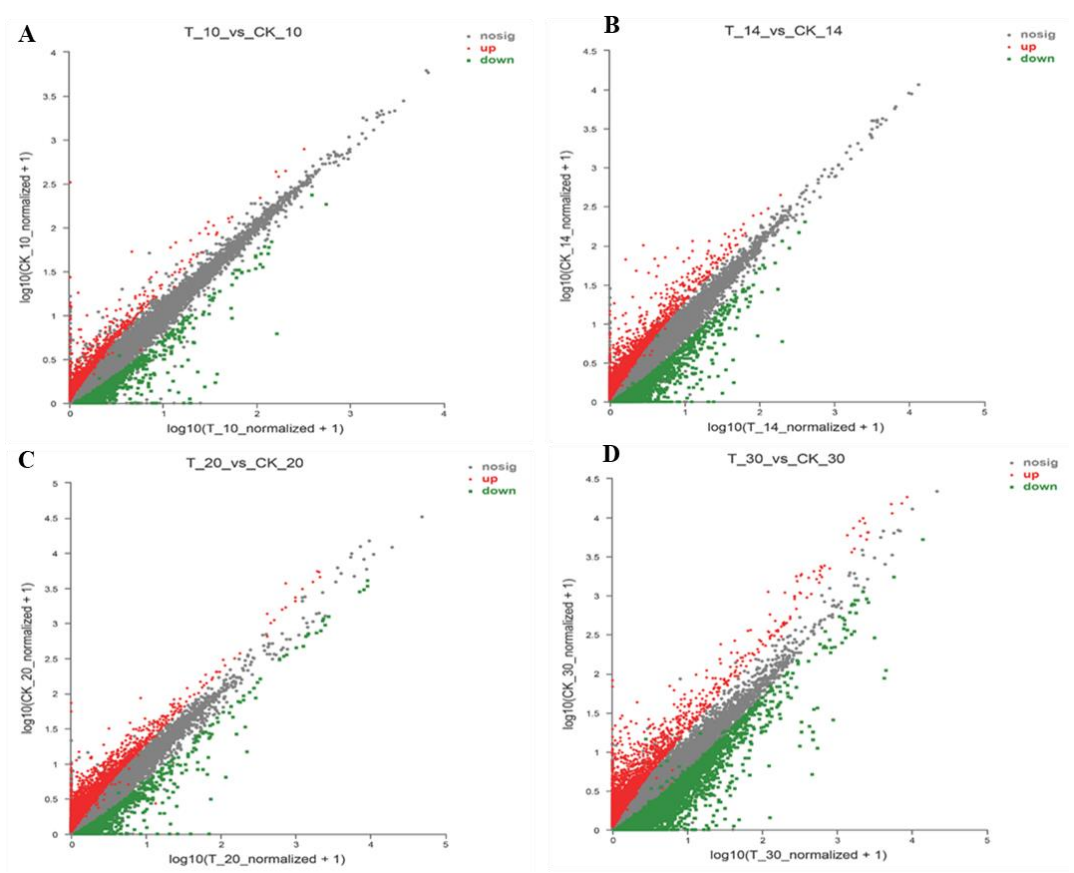
Volcano plots of DEGs in green versus red sectors. Data for all genes are plotted as log10 fold change (FC) versus the –log10 of raw p-value (pval). (**A**) Group T10 vs. CK10, (**B**) Group T14 vs. CK14, (**C**) Group T20 vs. CK20, and **(D**) Group T30 vs. CK30. Points outside the gray area indicate >100-fold-differences in expression. Green points indicate down-regulated genes and red points indicate up-regulated genes in each contrast, detail given in Supplementary file S1, sheet 3 (Table S1).

For comprehensive analysis, to understand the comparative difference between different groups in association with DEGs, which controlled the protein procession within the ER, all four constructed groups “T10 vs. CK10, T14 vs. CK14, T20 vs. CK20, and T30 vs. CK30” were analyzed. The similarity index of DEGs between all used groups is provided in Figure 10. There were 1838 genes reported in “T10 vs. CK10” in comparison with “T14 vs. CK14” were 4137. The minimum number of DEGs were seen in “T10 vs. CK10” in comparison with the three other groups. This suggested the primary involvement of the “cell division” phase of 10 DPA in protein quality control of the ER. Further, to identify the transcript level of DEGs in comparison with different developing stages of both used wheat samples, the transcript level of each gene in all four groups was compared and filtered with |log10 (TPM)|> = 1 and FDR < 0.001; for further detail, see Supplementary File S1, sheet 4.

### 2.5. Putative Genes Related to Protein Quality Control

To further investigate the role of ER-associated protein folding, sorting, and degradation, at different stages of grain development in wheat, the candidate genes related to cell wall organization or biogenesis, protein transporter activity, the structural constituent of ribosomes, protein folding, unfolded protein, intracellular protein transport, GTPase activity, ATPase activity, vesicle-mediated transport, N-glycan processing (ER), intracellular protein transport (COPI vesicle coat), intra-Golgi vesicle-mediated transport, mismatch repair, retrograde vesicle-mediated transport, Golgi to ER, Full - Glutenin, protein serine/threonine kinase activity, beta-amylase activity, low-molecular-weight glutenin (LMW) subunit, glycogen (starch) synthase activity, and cysteine-type endopeptidase activity were identified according to the GO annotation and local TBLAST search. Only the genes with more than a two-fold change in FPKM within the developing grain of all selected stages are enlisted, with details given in (Supplementary File S4, sheets 1–5).

**Figure 11:**
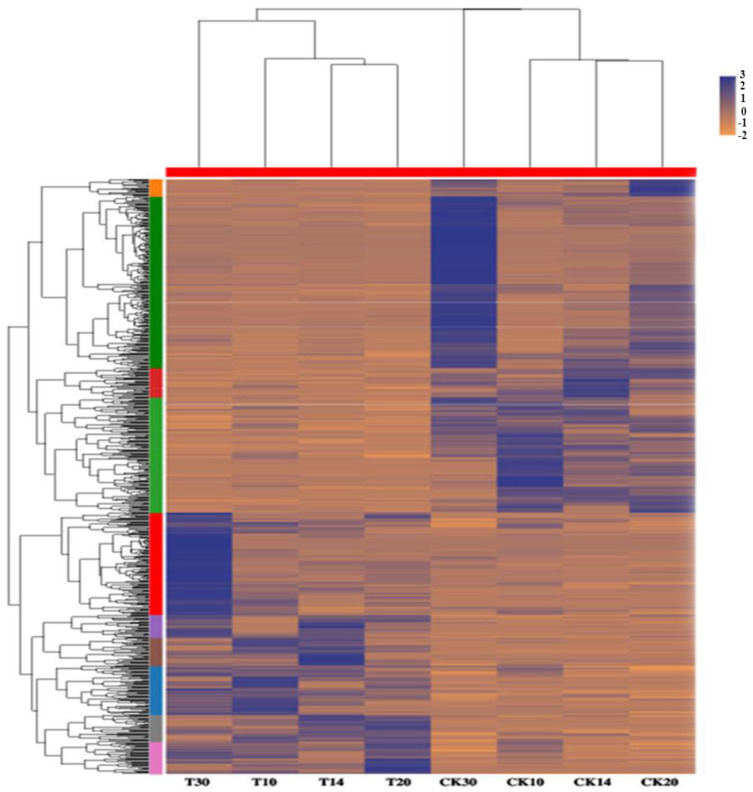
Hierarchical clustering analysis of all DEGs of all collected samples T10, T14, T20, T30, CK10, Ck14, Ck20, and CK30. A total of 696 genes were identified at *p* < 0.05 (ANOVA and Tukey HSD test); means ± SD. The color key represents the FPKM (Fragments per Kilobase of exon per Million fragments mapped)-normalized log2 transformed counts. Blue and Brown represent high and low expression, respectively. Each row represents a gene, with further detail given in Supplementary File S5.

**Figure 12:**
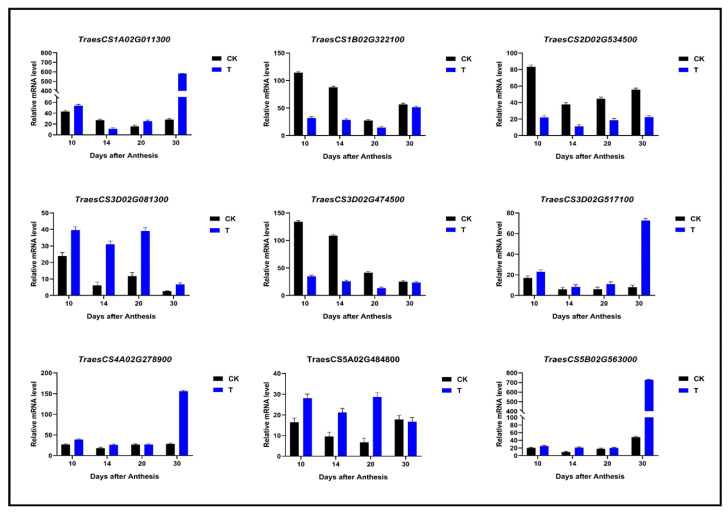
QRT-PCR analysis of 9 randomly selected mRNA expressions from transcriptome database, (T) Shaanong 33 (*Triticum aestivum* L.) and CK (*Triticum aestivum* L.), at 10, 14, 20, and 30 DPA. Data are means of three biological replicates (n = 3); significant differences at *p* < 0.05 (ANOVA and Tukey HSD test); means ± SD.

A total of 219 and 175 genes were identified to be linked with protein folding, unfolding, a structural constituent of ribosomes (ER), and intracellular protein trafficking such as GTPase activity and ATPase activity, respectively, given in Supplementary File S4, sheet 1. The genes regarding the defense mechanism are mainly involved in response to the biotic stimulus and disease resistance protein RPP13 (Supplementary File S4, sheet 3), and intracellular-trafficking-, secretion-, and vesicular-transport-related DEGs (Supplementary File S1, Sheet 3 (Table S1) & S4, sheet 2). The 84 genes identified here encode for grain quality characteristics such as grain hardness (protein serine/threonine kinase activity), starch synthesis, and viscoelasticity (cysteine-type endopeptidase activity) (Supplementary File S4 and sheet 4). Further, all the expressed genes in all collected samples of Shaannong 33 and CK are shown in Figure 11, with detail given in Supplementary File S5. There were a total of 696 DEGs in all collected samples, from which *TraesCS1A02G011300*, *TraesCS1B02G017000*, and *TraesCS1B02G276200* showed a two-fold transcript level in T30 samples. *TraesCS4A02G316000* and *TraesCS5D02G551600* reported an optimum two-fold transcript level in T10. In comparison, *TraesCS4A02G316000* reported an optimum transcript level, with further detail shown in Figure 11, Supplementary File S5. The expression profile of all genes involved in protein trafficking and protein folding and unfolding were well consistent with FPKM values from different developing stages (Figure 11). The expression profiles of all genes involved in protein trafficking and protein folding and unfolding were well consistent with FPKM values from different development stages (Figure 11). *TraesCS1A02G133100* was associated with GO: 0006457 (20 DPA) and T30 (30 DPA). This suggested intracellular protein quality control within the ER. GTPase activity has been approved as an important regulator in gluten quality characteristics, through controlling ER-to-Golgi vesicle trafficking [59,60,61,62,63]. *TraesCS1A02G137213* linked with GO: 0043547 (positive regulation of GTPase activity) showed a higher expression at 10 DPA (cell division phase), which decreased at 30 DPA. Similarly, *TraesCS1A02G131500* showed a higher GO: 0007264 (mall-GTPase-mediated signal transduction) expression at 10 DPA, which then decreased with exposure to increasing DPA. *TraesCS1A02G060700* suggested a linkage with translation, ribosomal structure, and biogenesis expression decrease until 30 DPA. *TraesCS1A02G092900* gene showed that GO: 0003735 (structural constituent of ribosome) expression increases with increasing development, a putative regulator during protein processing within the ER quality controlling process. *TraesCS1A02G126800* showed that the GO: 0080163 (regulation of protein serine/threonine phosphatase activity) expression level was medium when exposed to grain development stages. Defense-mechanisms-related putative genes such as *TraesCS1A02G081300* showed optimum expression at 30 DPA. A total of nine randomly selected genes were validated through RT-qPCR analysis (Figure 12).

## 3. Discussion

In the developing endosperm of bread wheat (*Tritium aestivum* L.), seed storage proteins are produced on the rough Endoplasmic Reticulum (rER) and transported to protein bodies, specialized vacuoles for the GSP (Gluten) synthesis. The GSP is the main source of gluten protein synthesis within endosperm, governing its end-use value [27,28,30,31,32,33,34,35,36,64]. However, the underlying mechanism of protein quality during ER-to-Golgi trafficking, specifically folding and unfolding, is still a challenging factor in molecular terms [65]. The present study conducted a compressive analysis by using a deep transcriptomic survey at four critical stages (10 DPA, 14 DPA, 20 DPA, and 30 DPA), to observe systematic changes and the potential mechanism associated within the developing grain of high-quality characteristics of wheat cultivar Shaannong33 (*Triticum aestivum* L.), in association with its low-quality sister line (CK). The peak mitotic division within starchy endosperm occurs after 10 DPA in maize, after 12 in barley, and in wheat, it remains until 16 DPA [66,67,68,69,70]. A sum total of 125,729 DEGs were retrieved at all selected stages and 94,972 genes were classified in all four groups (T10 vs. CK10, T14 vs. CK14, T20 vs. CK20, and T30 vs. CK30) for further study. They were mainly involved in translation, ribosomal structure and biogenesis, intracellular trafficking, secretion, vesicular transport, defense mechanism, serine synthesis, cell wall modeling, carbohydrate metabolism, starch and sucrose metabolism, and many unknown functions (Table 3 and Figure 5, Figure 6, Figure 7 and Figure 8), which were also widely studied in other plant species. A number of studies had confirmed that the rule of the effects of GSP trafficking between the ER to Golgi body is linked with grain processing quality characteristics, especially during grain filling [27,28,65,70,71,72]. Here, we identified 315 DEGs in pathway id (map04141) that are linked with protein processing within the ER in the secondary category described as folding, sorting, and degradation, linked with GSPs degradation (Figure 9 and Table 3). The maximum translation, ribosomal structure, and biogenesis took place within T10 vs. CK10, T14 vs. CK14, and T20 vs. CK20 suggested optimum mitotic cell division, as previously reported by [68,69,70,71,72,73]. Further, we found that protein folding, sorting, and degradation-associated genes were enriched within 10 DPA-20 DPA. This might indicate the systematic role in quality control within ER to Golgi bodies, resulting in the enhancement of GSP quality improvement with storage protein activator (SPA). The SPA as a transcriptional regulator plays a significant role in GSP trafficking, ultimately defining dough viscoelasticity and grain hardness [57,73,74]. The 12 cysteine residues have a prominent effect on HMW-GS subunit “5 + 10” (more possible disulfide bonds in its chemical structure), leading to higher strength compared to the HMW-GS subunit (2 + 12), which possesses 11 cysteines [74,75]. Therefore, we suggested the significant number of genes in association with serine/threonine kinase activity, enlisted in Supplementary File S4. *TraesCS1A02G148428* showed a 63.71-fold expression level at 10 DPA, which was recognized in GO: 0006535 (cysteine biosynthetic process from serine), suggesting the putative role in increasing tensile resistance of the used wheat cultivar “Shaanong 33”. It further signifies the role of GSP trafficking to the ER. Furthermore, the specific stage of grain development could be helpful for further investigation and could open a new venue toward an ER protein quality control mechanism.

The ER is the largest organelle in the cell and is a major site of protein synthesis and transport, protein folding, calcium storage, and carbohydrate metabolism [58,59,76,77]. The maximum activity of starchy endosperm development took place until 16 DPA [31,56,78,79]. The transcript driving the progression of endosperm development was differently expressed at different stages of harvest [9,76,79], as shown in Figure 11. The 60–80% GSP consists of gluten, developed within starchy endosperm. It determines the dough cohesiveness and viscoelasticity [9,74,80,81], which is a highly recommended wheat for bread and Chinese yellow alkaline noodles making. The extensive role of GSP highly endorses it for further extensive studies. In addition, the dynamic role of quantitative genetic variation in GSP composition has been reported for wheat [3,9,39,40,82]. In this study, we detected 315 DEGs encoding GSPs translocation in association with map0414, shown in Figure 9, Supplementary File S4 (sheets 1–4). They trigger protein processing pathways to maintain a homeostasis of the total amount of protein per grain [75,82,83]. The Rab GTPase is best characterized in *Arabidopsis thaliana* for its key involvement in specifically the trafficking mechanism. It works as a molecular switch to drive the transport of vesicles between membranous compartments. The motor proteins within the membrane regulate vesicle and compartment motility [28,29,30,33,61,62,84]. Hence, different Rab sub-clades have been classified in protein vesicle bodies trafficking to the Golgi apparatus and vacuole [85]. Consistent with the previous successful work, the present study also reported 177 DEGs in protein vesicle bodies trafficking to Golgi apparatus (Supplementary File S4, sheet 2), from which *TraesCS1A02G223800* showed a 651.31-fold expression at 14 DPA, linked with GO: 0043547 functional association of positive regulation of GTPase activity. Further, at 14 DPA, the second major transition stage is where starch and seed storage proteins accumulate within cells to make semi-solid endosperm [56,79,86]. This suggested the role of storage protein activator SPA, in the key functional mechanism to maintain a homeostasis supply of nutrients between the maternal cells and the starchy endosperm, and linked with the serine-to-cysteine biosynthesis process. Rab GTPase has been approved in different quality characteristics of mango, and others [63,87]. Therefore, further investigation is needed to improve our knowledge. However, the current study could lead to a specific stage and candidate gene in association with protein processing within the developing grain.

## 4. Materials and Method

### 4.1. Plant Materials

Shaannong 33 is a newly released high-quality, strong gluten wheat variety that has been approved in Shaanxi Province and introduced in Henan Province, China [88,89]. A summary and details of Shaannong 33 are shown in Table 4, and the sister line of Shaannong 33, used as the control (CK), reported with low-quality traits, given in Table 1, represents three-year field trails. The plant material was planted in a 2 m row length, the plant-to-plant distance was 0.25 m, and each line was comprised of 40 plants, in three biological replicates [88,89,90,91], grown at Northwest Agriculture and Forestry University (Field station) under non-stressed conditions from October 2019 to 2020. The main individual spikes were tagged at the first day of anthesis. The tagged spikes were harvested at four main seed developmental stages, i.e., 10 DPA, 14 DPA, 20 DPA, and 30 DPA, immediately frozen in liquid nitrogen, and stored at −80 °C. The embryos of these samples were used for RNA extraction to isolate the entire RNA expressed in both used planted materials.

### 4.2. RNA Extraction

Total RNA was extracted from grains at 10, 14, 20, and 30 DPAs of Shaannong 33 and the control with three biological replicates by using Trizol Reagent according to the manufacturer’s instructions [88,89,92] (Invitrogen, Carlsbad, CA, USA), and genomic DNA was removed using DNase I (TaKara). Then, RNA quality was determined by the 2100 Bioanalyser (Agilent) and quantified using the ND-2000 (NanoDrop technologies). Only a high-quality RNA sample (OD260/280 = 1.8~2.2, OD260/230 ≥ 2.0, RIN ≥ 6.5, 28 S: 18 S ≥ 1.0, >10 μg) was used to construct the sequencing library [91]. For each of the samples at 10 DPA, 14 DPA, 20 DPA, and 30 DPA, three replicates of each time point were performed for the RNA-seq. 

### 4.3. Library Preparation and Illumina Hiseq4000 Sequencing

There were 24 RNA-seq transcriptome libraries that were prepared following the TruSeqTM RNA sample preparation Kit from Illumina (San Diego, CA, USA) using 5 µg of total RNA. First, messenger RNA was isolated according to the polyA selection method by oligo (dT) beads and then fragmented by fragmentation buffer. Secondly, double-stranded cDNA was synthesized using a Superscript double-stranded cDNA synthesis kit (Invitrogen, Carlsbad, CA, USA) with random hexamer primers (Illumina). Then, the synthesized cDNA was subjected to end-repair, phosphorylation, and “A” base addition according to Illumina’s library construction protocol. Libraries were size-selected for cDNA target fragments of 200–300 bp on 2% low-range ultra-Agarose followed by PCR amplified using Phusion DNA polymerase (NEB) for 15 PCR cycles. After quantification by TBS380, the paired-end RNA-seq sequencing library was sequenced with the Illumina HiSeq 4000 (2 × 150 bp read length) [91,92].

### 4.4. Read Mapping

The raw paired-end reads were trimmed and quality-controlled by 11 October 2021 SeqPrep (https://github.com/justjohn/SeqPrep) and 11 October 2021 Sickle (https://github.com/najoshi/sickle) with default parameters. Then, clean reads were separately aligned to reference *Triticum_aestivum* version: iwgsc_refseqv1.0 source: http://www.wheatgenome.org/News/Latest-news/All-IWGSC-reference-sequence-resources-now-publicly-available-at-URGI with the orientation mode using TopHat 2.0.12 (Johns Hopkins University, Baltimore, Maryland) (http://tophat.cbcb.umd.edu/.version2.0.0) [93] software. The mapping of a criterion of bowtie was as follows: sequencing reads should be uniquely mated to the genome, allowing up to 2 mismatches, without insertions or deletions. Then, the region of the gene was expanded following depths of sites, and the operon was obtained. In addition, the whole genome was spilt into multiple 15 kbp windows that share 5 kbp. New transcribed regions were defined as more than 2 consecutive windows without overlapped regions of the gene, where there were at least 2 reads mapped per window in the same orientation. 

### 4.5. Differential Expression Analysis and Functional Enrichment

A total of 24 RNA samples from Shaannong 33 and CK samples at four spike developmental stages (10, 14, 20, and 30 DAA) were sent to Shanghai Majorbio Bio-Pharm Technology Co., Ltd. (Shanghai, China) using paired-end sequencing on an Illumina HiSeq PE150 platform Shanghai Meiji Biomedical Technology (Shanghai, China) for RNA-sequencing and transcriptome assembly using previously described protocols [91,92]. Fragments per kilo-base of transcript per million mapped reads (FPKM) values were used to calculate transcript abundance in Shaannong 33 and CK collected samples. 21 October 2021 RSEM (http://dewelab.biostat.wisc.edu/rsem/) [94] was used to quantify gene abundances. A summary of the sequencing statistics for reads and bases obtained in each sample and statistics for the mapping of clean reads from each sample to the reference genome are given in Supplementary File S1. Further, the significance of differentially expressed genes (DEGs) was analyzed using R Statistical package software at 30 October 2021, EdgeR (empirical analysis digital gene expression in R, http://www.bioconductor.org/packages/2.12/bioc/html/edeR.html), a published method [95]. The biological processes associated with each gene in each sample were evaluated using Gene Ontology (GO) analysis 1 November 2021 (http://www.geneontology.org/). KEGG pathways with corrected *p*-values < 0.01 were considered to be significantly enriched (http://www.genome.jp/kegg/) [96].

### 4.6. New Isoforms Prediction

The TOpHat-Cufflinks pipeline was used to predict gene isoforms from our RNA-seq data. In 1 January 2022 TopHat (http://tophat.cbcb.umd.edu/, version 2.0.0) [93], the option “min-isofom-fraction” was disabled; instead, “coverage-search”, “butterfly-search”, and “microexon-search” were used. The expected fragment length was set to 200 bp and the “small-anchor-fraction” was set to 0.08, which requires at least 8 bp on each side of an exon junction for our 100-bp RNA-seq data. Cuffcompare was used to compare and merge the reference annotation and the isoform predictions.

### 4.7. Alternative Splice Events Identification

All the alternative splice events that occurred in our sample were identified by using the recently released program Multivariate analysis of 1 December 2021 Transcript Splicing (MATS, http://rnaseq-mats.sourceforage.net/) [97]. Only the isoforms that were similar to the reference or comprised novel splice junctions were considered, and the splicing differences were detected as exon inclusion, exclusion, alternative 5’, 3’, and intron retention events.

### 4.8. Gene Expression Level Validation by qRT-PCR

Total RNA was isolated by using a total RNA kit (TIAGEN, Beijing, China) following the manufacturer’s instructions, followed by [88,89]. For the complementary DNA (cDNA) synthesis, 2 mg of total RNA was reverse-transcribed using a Prime-Script TM II First-Strand cDNA synthesis Kit (TakaRa, Dalian, China). The quantitative real-time PCR (qRT-PCR) analysis was performed with a Light Cycle 480 II system (Roche, Basel, Switzerland) using an SYNR Premix Ex TaqTM kit (TakaRa, Dalian, China). The UBI-eq gene was used as an internal control. The primer sequences used for the PCR are given in (Supplementary File S5, sheet 2). Three replicates were made for three separate RNA extracts from the three samples.

## 5. Conclusions

**Figure 13:**
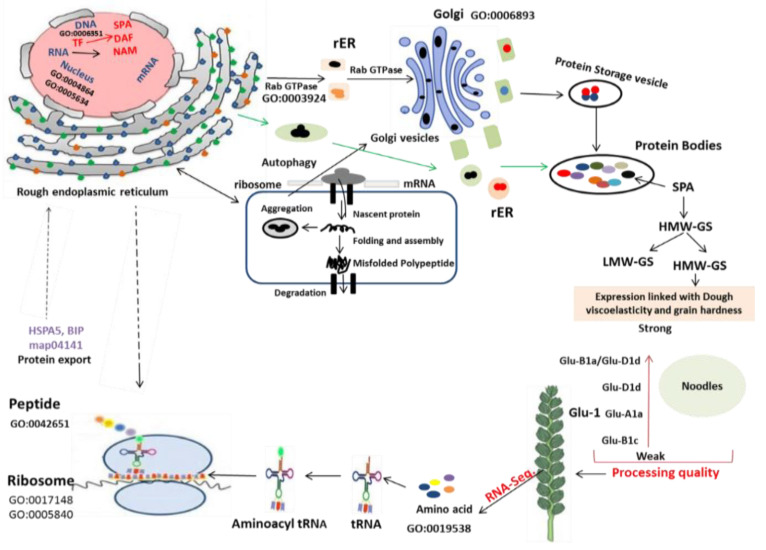
Potential mechanism of protein synthesis and trafficking from ER to Golgi body and ultimate linkage with processing quality characteristics in wheat grain.

The accumulation of protein bodies is taken either directly within the lumen of the ER or through the Golgi apparatus into the vacuole [27,28,29,30,31,59,60,61,62,63,70,98]. Here, we proposed a model through combining our results and previous successful works, which could be used to test how trafficking of GSP within the developing grain improves the wheat grain end-product quality (Figure 13). This proposal begins with the expression of the translation-, ribosomal-structure- and biogenesis-, and protein-folding- and unfolding-related DEGs. The TFs bind with the promoter region of a suite of genes, improving storage proteins and protein synthesis regulator proteins in wheat grains. Thus, the composition changes within the ribosome lead to a higher demand for protein synthesis and more molecular chaperones assisting nascent proteins to fold properly and be stored. The abundances of chaperones and other storage proteins into the protein storage vacuoles with enhanced stability led to a broad increase in the abundance of HMW-GSs, further enhancing storage protein synthesis and deposition, and leading to a significant improvement in the dough quality with changes in gluten composition. Therefore, this study suggested the identification of putative DEGs that could contribute to enhanced protein content, at different stages of DPA in wheat grains.

## Figures and Tables

**Table 1:** Analysis of variance for quality parameters of the used material in this study.

Variety	Flour Rate	Settlement Index	Water Absorption	Formation Time	Max. Tensile Resistance
**Shannong 33**	73.3%	66 mL	58.7	15.6	1038
**Sister line (C.K)**	71%	27.6 mL	63	3.1	358

**Table 2:** Summary of sequencing outcomes from grain enlargement stage.

Sample	Raw Reads	Raw Bases	Clean Reads	Clean Bases	Error Rate (%)	GC%	Q20 (%) ^a^	Q30 (%) ^b^	Total Mapped
**T10**	52,242,026	7,888,545,926	51,306,098	7,650,889,193	0.0248	54.64	98.03	94.4	45,136,960 (87.98%)
**T14**	58,357,186	8,811,935,086	57,143,490	8,473,951,442	0.0254	53.14	97.75	93.89	45,153,078 (79.02%)
**T20**	49,861,908	7,529,148,108	48,289,034	7,121,352,201	0.0268	52.91	97.17	92.6	37,102,266 (76.83%)
**T30**	49,553,172	7,482,528,972	48,697,252	7,236,265,313	0.025	54.35	97.96	94.25	37,230,131 (76.45%)
**CK10**	52,674,730	7,953,884,230	51,814,526	7,742,996,821	0.0246	55.02	98.11	94.59	46,754,208 (90.23%)
**CK14**	52,215,230	7,884,499,730	51,232,492	7,611,938,755	0.0253	53.75	97.8	94	43,115,178 (84.16%)
**CK20**	48,982,074	7,396,293,174	48,113,392	7,143,015,218	0.0252	52.64	97.8	94.03	34,274,380 (71.24%)
**Ck30**	51,353,948	7,754,446,148	50,053,338	7,410,668,772	0.0255	53.85	97.7	93.8	35,435,811 (70.8%)

^a^ Q20 indicates a quality score of 20, a 1% chance of error, and 99% confidence. ^b^ Q30 indicates a quality score of 30, a 0.1% chance of error, and 99.9% confidence.

**Table 3:** Summary of DEGs related to ER protein quality control pathways based on the KEGG database of developing grain at all selected stages.

Pathway Type	Pathway ID	DEGs	Up/Down (Numbers of Genes)	Gene ID
Cell wall synthesis (ER or Golgi cellular components)	map04714	Up	4	*TraesCS1A02G133900, TraesCS1A02G116200* *TraesCS1A02G124600, TraesCS1A02G137216*
structural constituent of ribosome (ER or ER cellular components)	map03010	Up	6	*TraesCS1A02G092900, TraesCS1A02G105400, TraesCS1A02G137294, TraesCS1A02G137403, TraesCS1A02G137409, TraesCS1A02G137505*
Protein folding (During ER protein processing)	map03060	Up	8	*TraesCS1A02G112400, TraesCS1A02G138121, TraesCS1A02G133100, TraesCS1A02G133700, TraesCS1A02G124400, TraesCS1A02G145979, TraesCS1A02G099300, TraesCS1A02G137277, TraesCS1A02G137424, TraesCS1A02G137449*
ATPase activity, GTPase activity (ER to Golgi trafficking)	map03060	Up	18	*TraesCS1A02G126900, TraesCS1A02G131500, TraesCS1A02G144411, TraesCS1A02G144222, TraesCS1A02G143755, TraesCS1A02G143642, TraesCS1A02G143622, TraesCS1A02G143335, TraesCS1A02G143297, TraesCS1A02G142230, TraesCS1A02G137242, TraesCS1A02G137259, TraesCS1A02G137367, TraesCS1A02G137536, TraesCS1A02G137607, TraesCS1A02G137612* *TraesCS1A02G137736, TraesCS1A02G137782*
vascular transport (ER to Golgi vesicle-mediated transport)	map03060	Up	8	*TraesCS1A02G144928, TraesCS1A02G143916, TraesCS1A02G106000, TraesCS1A02G133100, TraesCS1A02G137356, TraesCS1A02G137364, TraesCS1A02G137405, TraesCS1A02G151679*
Protein quality control		Up	4	*TraesCS1A02G137200, TraesCS1A02G150621, TraesCS1A02G151217, TraesCS1A02G108100*

**Table 4:** Detail of the wheat varieties used in this study.

Variety	Parentage	Year (Releasing Committee)	Developed by	Area of Adoption	Source
**Shaannong 33**	Xinmai 18sp-28-14/Shannong 981sp-12-16	2011-Shaanxi Provincial crop variety appraisal committee	Wang Chengshe, Liu Luxiang, Zou Shufang, Xu xitang Chen, Guangdou, Xie Yangzhou	Shaanxi, and Henan Province China	NWAFU

## Data Availability

The main data supporting the results of this article are included within the article and the provided in Supplementary Materials.

## Supplementary Materials

The supporting information can be downloaded at: https://www.mdpi.com/article/10.3390/ijms232314851/s1.

## Abbreviations

ER: Endoplasmic reticulum; GO: gene ontology; HC: high confidence; IWGSC: International Wheat Genome Sequencing Consortium; QTL: quantitative trait loci; ROS: reactive oxygen species; RT-qPCR: real-time quantitative PCR. Statement: All methods and material used are complied with relevant institutional, national, and international guidelines and legislation in the methods section of the manuscript.
