# Supplementary material for: Transcriptome Analysis Reveals Potential Mechanism in Storage Protein Trafficking within Developing Grains of Common Wheat
Source: Int J Mol Sci. 2022 Nov 27;23(23):14851. doi: 10.3390/ijms232314851 (PMC9738083; doi:10.3390/ijms232314851)
Supplement: Supplementary file 1 [file caption.pdf › caption.pdf]

Supplementary file 1:

Sheet 1: Overview of reads processing and reference genome mapping of all samples.

Sheet 2: Figure 5. KEGG pathway enrichment analysis for 127 candidate genes.

Sheet 3: Table S1. Differently expressed gene in comparison with different grain development stages.

Sheet 4: Comparison of DEGs of all four groups of developing stages.

Sheet 5: Overview of reads processing and reference genome mapping from all four group libraries.

Supplementary file 2: Figure 6. Clusters of KEGG function classification of DEGs.

Sheet 1: Group 1 (T10 vs. CK10).

Sheet 2: Group 2 (T14 vs. CK14).

Sheet 3: Group 3 (T20 vs. CK20).

Sheet 4: Group 4 (T30 vs. CK30).

Supplementary file 3: Clusters of GO function classification of DEGs.

Sheet 1: Group 1 (T10 vs. CK10).

Sheet 2: Group 2 (T14 vs. CK14).

Sheet 3: Group 3 (T20 vs. CK20).

Sheet 4: Group 4 (T30 vs. CK30).

Supplementary file 4: Figure 9. KEGG pathway analysis DEGs in association with protein trafficking pathway (ko04141).

Sheet 1: Translation-, ribosomal-structure- and biogenesis-, and protein-folding- and protein-unfolding-related group of DEGs.

Sheet 2: Intracellular-trafficking-, secretion-, and vesicular-transport-related DEGs.

Sheet 3: Disease and stress resistance DEGs.

Sheet 4: Signal transduction mechanisms and gluten sub-units controlling DEGs.

Sheet 5. Group of DEGs located in plasma membrane and others involved in transportation.

Supplementary file 5: Figure 11. Hierarchical clustering analysis of all DEGs of all collected samples.

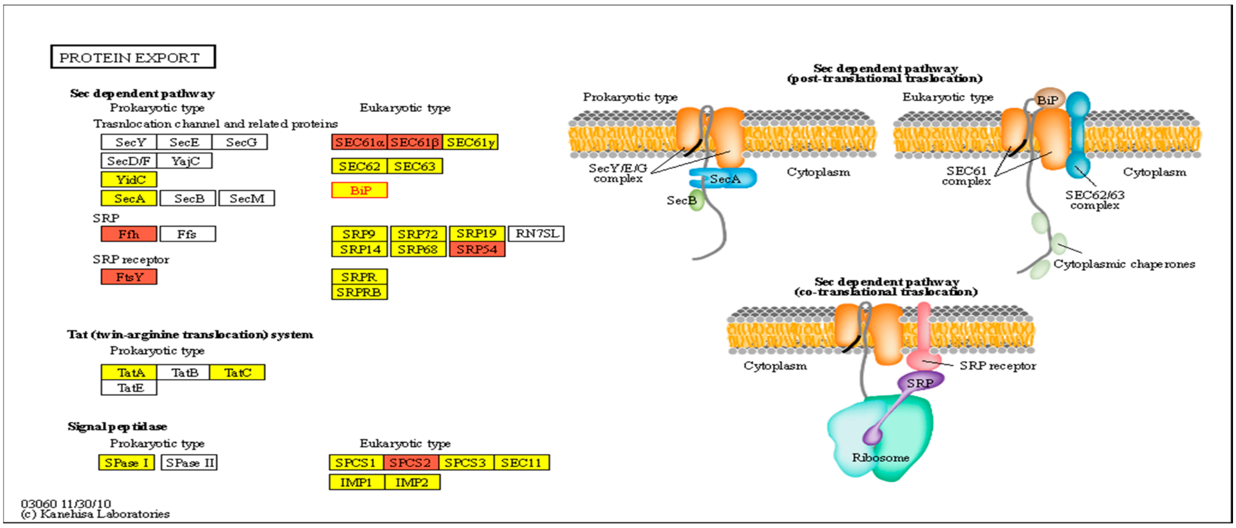

Figure S1 Protein export at different stages of grain development.
